# CT-Assessed Body Composition as Predictor of Post-Operative Complications in Lung Cancer Patients

**DOI:** 10.3390/cancers18030431

**Published:** 2026-01-29

**Authors:** Stefania Rizzo, Francesco Petrella

**Affiliations:** 1 Clinic of Radiology EOC, Via Tesserete 46, 6900 Lugano, Switzerland; 2 Faculty of Biomedical Sciences, Università della Svizzera Italiana (USI), Via G.Buffi 13, 6900 Lugano, Switzerland; 3 Department of Thoracic Surgery, Fondazione IRCCS San Gerardo dei Tintori, 20900 Monza, Italy; francesco.petrella@irccs-sangerardo.it; 4 Department of Oncology and Hemato-Oncology, University of Milan, Via Festa del Perdono 7, 20122 Milan, Italy

**Keywords:** body composition, sarcopenia, computed tomography (CT), lung cancer surgery, postoperative complications

## Abstract

Body composition, specifically the quantification of skeletal muscle and adipose tissue using preoperative computed tomography (CT) imaging, is a clinically significant predictor of postoperative complications after lung cancer surgery. The main features of CT-derived body composition analysis are: skeletal muscle index, muscle density, adipose tissue quantification and automated or semi-automated segmentation. Low skeletal muscle mass (sarcopenia) independently increases the risk of perioperative complications, including respiratory complications, and is associated with longer hospital length of stay and worse long-term survival. Sarcopenic obesity—characterized by low muscle mass in the context of high adiposity—further elevates complication risk and prolongs recovery. CT-derived measures such as muscle cross-sectional area, muscle density, and adipose tissue distribution (visceral, subcutaneous, and intramuscular) provide more precise risk stratification than BMI alone. Skeletal muscle area and density are inversely correlated with postoperative complications and recurrence risk; patients with lower muscle mass and density experience more adverse outcomes. In men, age and reduced skeletal muscle area are particularly strong predictors of complications after pneumonectomy. Obesity, when not accompanied by sarcopenia or myosteatosis, may confer a survival advantage—the so-called “obesity paradox”—but this protective effect is lost in patients with low muscle mass or poor muscle quality. Systemic inflammation and nutritional status further modulate the impact of body composition on surgical risk. This review highlights the critical role of CT-derived body composition analysis in predicting postoperative outcomes following lung cancer surgery.

## Simple Summary

Patients with lung cancer can have very different amounts of muscle and body fat, and these differences may affect how well they recover from surgery. Traditional measures like body weight or body mass index do not fully capture these risks. The authors aim to show how routine computed tomography scans taken before surgery can be used to measure muscle and fat more accurately and identify patients who are more likely to develop complications. In particular, low muscle mass, especially when combined with high body fat, increases the risk of breathing problems, longer hospital stays, and poorer long-term outcomes. These findings may help researchers and clinicians to better estimate the surgical risks, to improve patient selection, and to encourage future studies on nutrition, exercise, and personalized care to improve recovery after lung cancer surgery.

## 1. Introduction

Lung cancer remains the leading cause of cancer-related mortality worldwide, with approximately 2.48 million new cases and 1.8 million deaths reported in 2022 [1]. Surgical resection continues to be the cornerstone of curative-intent treatment for patients with early-stage and select locally advanced non-small-cell lung cancer (NSCLC), with lobectomy representing the gold standard approach [2]. Despite advances in minimally invasive surgical techniques and perioperative care, postoperative morbidity rates following lung resection remain substantial, ranging from 18.5% to 30.8%, with mortality rates of 2.6% in contemporary series [3]. These complications not only affect immediate surgical outcomes but they also delay adjuvant therapy, prolong hospital stays, and adversely affect long-term survival.

Traditional preoperative risk assessment in lung cancer surgery has relied primarily on pulmonary function testing (forced expiratory volume in 1 s and diffusion capacity for carbon monoxide), cardiopulmonary exercise testing, and clinical scoring systems such as the Thoracic Revised Cardiac Risk Index; the Revised Cardiac Risk Index (RCRI) [4,5]. While these measures provide valuable physiologic data, they may not fully capture the patient’s overall functional reserve and vulnerability to surgical stress. Body mass index (BMI), commonly used as a simple anthropometric measure, fails to distinguish between muscle mass and adipose tissue distribution, potentially misclassifying patients with normal or elevated BMI that harbour occult muscle depletion—a phenomenon known as sarcopenic obesity [6,7].

Computed tomography (CT)-based body composition analysis has emerged as a powerful, objective tool for preoperative risk stratification in surgical oncology [8]. Routine staging chest CT scans provide an opportunity for opportunistic assessment of skeletal muscle mass, muscle quality (radiodensity), and adipose tissue distribution, without additional radiation exposure or cost [9]. At thoracic vertebral levels (T5, T8, T10, T12) or the more commonly used level of the third lumbar vertebra (L3), cross-sectional imaging allows precise quantification of skeletal muscle area (SMA), skeletal muscle density and intramuscular adipose tissue, reflecting the fat infiltration of muscles. At the level of L3, the visceral adipose tissue (VAT) and subcutaneous adipose tissue (SAT) compartments are also usually assessed [10]. From the above mentioned measurements, further values can be calculated, such as the skeletal muscle index (SMI), corresponding to the SMA divided by square height; visceral adipose tissue index (VATI), corresponding to the VAT divided by the square height; subcutaneous adipose tissue index (SATI), corresponding to the VAT divided by the square height. These measurements correlate strongly with whole-body muscle mass and have demonstrated superior prognostic value compared to BMI across multiple cancer types [11].

In more detail, in lung cancer patients, emerging evidence indicates that CT-assessed sarcopenia—defined by low muscle mass and/or low muscle density—independently predicts increased perioperative complications, prolonged hospital length of stay, and reduced overall and cancer-specific survival following anatomic lung resection. Recent multicenter studies have shown that patients with sarcopenia face approximately 2.5-fold higher odds of perioperative complications and significantly worse long-term outcomes [9,12]. Furthermore, composite body composition profiles incorporating myosteatosis, sarcopenia, and visceral obesity appear to provide enhanced risk stratification beyond individual parameters alone [13]. The advent of artificial intelligence-assisted automated segmentation tools now enables rapid, reproducible body composition analysis that can be seamlessly integrated into clinical workflows, facilitating translation from research to routine practice [14].

Conventional preoperative risk assessment tools—such as the American Society of Anesthesiologists (ASA) Physical Status Classification, the Revised Cardiac Risk Index (RCRI), and the National Surgical Quality Improvement Program (NSQIP) risk calculator—largely depend on medical history, existing comorbidities, functional capacity, and basic laboratory results to predict perioperative risk, particularly for cardiovascular and general surgical complications. In contrast, computed tomography (CT)-based body composition analysis offers objective, quantitative evaluation of muscle mass, muscle quality (radiodensity), and fat distribution (visceral and subcutaneous) using standard preoperative imaging. These measures directly reflect sarcopenia, myosteatosis, and visceral obesity, which independently predict postoperative complications, prolonged hospital stays, and readmission across various surgical groups. CT-based analysis can therefore identify high-risk patients who may be overlooked by traditional screening methods, and multidimensional phenotyping (such as sarcopenic obesity) provides enhanced risk stratification for surgical morbidity, including infectious, wound-related, and major complications [14].

This review synthesizes the most current evidence on CT-assessed body composition as a predictor of postoperative complications in lung cancer patients, examining the methodological approaches, prognostic significance of specific body composition parameters, sex-specific considerations, and the potential for clinical implementation to optimize preoperative risk assessment and guide targeted interventions.

## 2. Search Strategy

This review outlines key aspects of CT-assessed body composition as predictor of post- operative complications in lung cancer patients. A comprehensive literature search was conducted using PubMed, Medline, and Google Scholar to identify relevant publications. The search strategy employed the following MeSH terms: “body composition”; “sarcopenia”; “computed tomography (CT)”; “lung cancer surgery”; “postoperative complications”. Eligible studies included those reporting patient demographics, clinical presentation, and management strategies related to our topic. We considered clinical trials, cohort studies, and case–control studies published in English prior to January 2026. Reference lists of selected articles were also manually reviewed to identify additional relevant studies. Exclusion criteria were opinion pieces, letters to the editor, abstracts, and preprints that had not undergone peer review.

## 3. Sarcopenia: Definition, Assessment and Clinical Impact

Sarcopenia represents a progressive and generalized loss of skeletal muscle mass and strength, leading to impaired physical function and increased risk of adverse outcomes. The most widely accepted diagnostic criteria, as updated by the European Working Group on Sarcopenia in Older People and the Asian Working Group for Sarcopenia, require both low muscle strength and low muscle mass for a definitive diagnosis [15]. The Global Leadership Initiative in Sarcopenia consensus further incorporates muscle-specific strength as a component [16]. Assessment by CT scan is a robust and objective method for quantifying muscle mass. The SMI, typically measured at the level of L3, is used to define sarcopenia based on established cutoffs [17]. In thoracic oncology, muscle area at the thoracic vertebrae (e.g., T10, T12) or pectoralis muscle on chest CT can serve as surrogates when abdominal imaging is unavailable, with good correlation to L3 measurements and approximation to the whole body muscle mass [18,19]. CT also allows assessment of muscle quality via radiation attenuation (density), which reflects fatty infiltration. Impact on surgical outcomes is substantial. Sarcopenia independently predicts increased risk of perioperative complications, prolonged hospital stay, higher morbidity, and worse overall and cancer-specific survival across multiple surgical populations, including gastrointestinal, hepato-bilio-pancreatic, thoracic, and orthopedic surgery [20,21]. Sarcopenic patients are more likely to experience major complications (Clavien-Dindo grade ≥ III), adverse discharge disposition, and reduced functional recovery postoperatively. Early identification via CT enables risk stratification and may inform prehabilitation strategies to mitigate these risks [22]. Sarcopenia is associated with significantly worse long-term survival after lung resection. Five-year overall survival rates are consistently lower in sarcopenic patients (ranging from 53% to 75%) compared to non-sarcopenic patients (61% to 91%), and multivariable analyses confirm sarcopenia as an independent predictor of mortality. The combination of sarcopenia and immunonutritional impairment further worsens prognosis and response to treatment for recurrence.

Preoperative assessment of sarcopenia using CT-based skeletal muscle index or functional measures (e.g., handgrip strength, peak expiratory flow rate) is feasible and recommended for risk stratification and perioperative management in lung cancer surgery. Early identification may guide prehabilitation strategies, including exercise and nutritional interventions, to mitigate risk [9,10,13].

## 4. The Role of CT Scan

CT scan is currently the most accurate and widely used imaging modality for opportunistic assessment of body composition in clinical and research settings [23].

The most common variables extracted by a CT scans, their definition and clinical relevance in lung cancer surgery are summarized in Table 1.

CT-derived measurements of muscle mass and adiposity are robust predictors of frailty, sarcopenia, and adverse outcomes in oncology, surgery, and critical illness. Recent advances include fully automated, artificial intelligence-driven segmentation tools that provide rapid, reproducible, and accurate quantification of muscle and fat compartments, facilitating clinical implementation and overcoming the limitations of manual analysis [8]. CT-based body composition analysis shows strong concordance with established modalities such as bioelectrical impedance analysis (BIA) and dual-energy X-ray absorptiometry (DEXA), with correlation coefficients often exceeding 0.9 for fat and muscle indices [24]. Technical parameters—such as contrast phase, tube current, slice thickness and reconstruction algorithms—can influence CT-based measurements, particularly muscle density, underscoring the need for standardized protocols to ensure reproducibility. While most clinical CT scans do not cover the entire body, validated algorithms can reliably estimate whole-body composition from regional scans (e.g., chest or abdomen) [25]. Although CT-based body composition analysis is increasingly recognized for its prognostic value and its potential to guide individualized therapy, especially in oncology and metabolic disease, consensus on standardized thresholds and reporting remains an area for ongoing research and development. Current consensus supports the use of single-slice abdominal CT at the third lumbar vertebra (L3) as the standard anatomical site for body composition assessment, with automated segmentation tools increasingly adopted for quantifying skeletal muscle, VAT and SAT [23]. The American Society for Parenteral and Enteral Nutrition, via the Global Leadership Initiative on Malnutrition (GLIM), recommends the SMI at L3 (muscle area/height^2^) as the preferred metric for muscle mass phenotyping in malnutrition diagnosis, especially in oncology and chronic disease populations [26]. Threshold values for sarcopenia and muscle quality have recently been meta-analyzed in healthy young adults: mean SMI values are approximately 54.6 cm^2^/m^2^ for men and 42.4 cm^2^/m^2^ for women, with T-score −2 cutoffs (analogous to osteoporosis criteria) at 36.3 cm^2^/m^2^ for men and 27.5 cm^2^/m^2^ for women; mean skeletal muscle density (SMD) is 47.4 HU for men and 43.6 HU for women, with T-score −2 cutoffs at 36.4 HU (men) and 28.1 HU (women) [27]. For prediction of adverse outcomes, sex-specific thresholds for muscle attenuation (density) have been proposed: 31 HU for men and 23 HU for women for all-cause mortality, with 90% specificity thresholds at 23 HU (men) and 13 HU (women) [28]. Technical parameters (contrast phase, tube current, slice thickness, reconstruction algorithm) must be standardized, as they significantly affect attenuation-based metrics, especially muscle density. Automated AI-based segmentation is validated and recommended for clinical implementation, with volumetric slabs around L3 potentially increasing resilience and reproducibility [29]. More recently, other tools able to segment the whole body to extract directly body composition measures, rather than estimates, has been introduced [30,31] as well as able to automatically select the correct slice at the level of L3 for extraction of body composition measures [32].

Clinical application is strongest in oncology, geriatrics, and metabolic disease, where CT-derived muscle and fat metrics are robust predictors of frailty, sarcopenia, and cardiometabolic risk. Routine use is recommended when CT imaging is already indicated for other reasons, minimizing additional radiation exposure and cost [33].

## 5. Prediction of Postoperative Complications in Lung Cancer Surgery

Prediction of postoperative complications in lung cancer surgery relies on multifactorial risk assessment, integrating patient characteristics, comorbidities, and surgical factors. Key predictors consistently identified in the medical literature include: older age (typically ≥65–70 years), male sex, and smoking history are associated with increased risk of complications, including pulmonary and cardiovascular events [34]. Chronic obstructive pulmonary disease (COPD) is a strong independent risk factor for both pulmonary and overall postoperative complications [35]. Poor pulmonary function, especially reduced diffusing capacity for carbon monoxide (DLCO), is a robust predictor of pulmonary complications; lower DLCO is associated with a twofold increased risk [35]. Comorbidities such as diabetes, hypertension, and previous malignancies further increase risk [36]. Surgical factors contribute to post-operative complications; in fact, more extensive resections (e.g., pneumonectomy), longer operative time, intraoperative blood transfusion, and conversion to open thoracotomy are associated with higher complication rates [35]. Moreover, preoperative chemotherapy or radiotherapy increases risk, particularly for pulmonary complications [37]. Nutritional status (e.g., low BMI, low prognostic nutrition index) and laboratory markers (e.g., lymphocyte–monocyte ratio, haemoglobin/red cell distribution width ratio) have been incorporated into recent predictive models [38]. Furthermore, BMI demonstrates a U-shaped relationship with risk. In fact, underweight patients (BMI < 18.5) have shown increased pulmonary complications and mortality, while moderate obesity (BMI 30–39.9) does not increase perioperative risk and may confer a protective effect, a phenomenon known as the “obesity paradox” [39]. Severe obesity (BMI ≥ 40) is associated with higher risk of major complications, but moderate obesity is linked to lower postoperative mortality and does not prolong hospital stay [39]. However, the protective effect of obesity is most evident in patients with preserved skeletal muscle mass and radiodensity [40]. In the context of the so-called “obesity paradox,” CT-based assessment of body composition helps clarify this phenomenon by identifying patients classified as obese by BMI who also have sarcopenia and therefore carry a higher risk. This distinction highlights how reliance on BMI alone can be misleading and may create an unwarranted perception of protection.

Machine learning models and nomograms integrating these variables (age, comorbidities, pulmonary function, surgical details, laboratory values) have demonstrated moderate to good predictive accuracy and may outperform traditional indices such as the Charlson Comorbidity Index [41]. Risk scores and prediction models should be used to guide perioperative management and patient selection, with targeted interventions for modifiable risk factors (e.g., smoking cessation, pulmonary rehabilitation, nutritional optimization) to reduce complication rates.

## 6. Association Between CT-Based Body Composition and Surgical Complications

CT-assessed body composition is a validated, independent predictor of postoperative complications in patients undergoing lung cancer surgery. Quantitative analysis of muscle mass, muscle quality (radiodensity), and adipose tissue compartments (subcutaneous, visceral, and intermuscular fat) can be performed on routine preoperative chest CT scans, typically at thoracic vertebral levels (T5, T8, T10) or lumbar level (L3) when available [42].

Low skeletal muscle mass (sarcopenia) and poor muscle quality (myosteatosis) are associated with increased risk of overall and respiratory postoperative complications, longer hospital length of stay, and worse survival after lung resection. Sarcopenic obesity—low muscle mass with high adiposity—further increases risk [42]. In men, decreased skeletal muscle area is particularly predictive of complications after pneumonectomy [18]. Muscle quantity and quality scores derived from CT imaging outperform traditional measures such as BMI in risk stratification, as BMI does not account for muscle-fat distribution or muscle quality [43]. Meta-analyses confirm that sarcopenia is associated with a more than twofold increased risk of perioperative complications and worse long-term survival [20]. CT-based body composition analysis is feasible using automated segmentation algorithms and can be integrated into preoperative risk models alongside clinical variables (age, pulmonary function, comorbidities) to improve prediction accuracy. These assessments are objective, reproducible, and can be performed on routine staging CT scans, facilitating individualized risk stratification and perioperative planning [9].

The latest evidence demonstrates that preoperative exercise training and targeted nutritional support are the most effective interventions to improve body composition and reduce postoperative complications in patients undergoing lung cancer surgery. In fact, preoperative exercise programs—typically combining aerobic, resistance, and respiratory muscle training—have consistently shown a significant reduction in postoperative complications (risk reduction up to 50%), shorter hospital stays, and improved pulmonary function and exercise capacity in randomized controlled trials and meta-analyses [44,45]. These programs are generally delivered over 2–4 weeks prior to surgery, with moderate intensity and frequency tailored to patient tolerance and baseline fitness. The American Society of Clinical Oncology recommends structured preoperative exercise for patients with lung cancer to reduce postoperative pulmonary complications and length of stay [46]. Nutritional interventions, especially when targeted to patients with malnutrition or sarcopenia identified by CT body composition analysis, are associated with improved muscle mass, strength, and reduced complication rates. Strategies include individualized dietary counseling, high-protein oral supplements, and, when indicated, enteral or parenteral nutrition. Multimodal prehabilitation—combining exercise and nutrition—further enhances functional reserve, nutritional status and perioperative outcomes [47].

**Figure 1:**
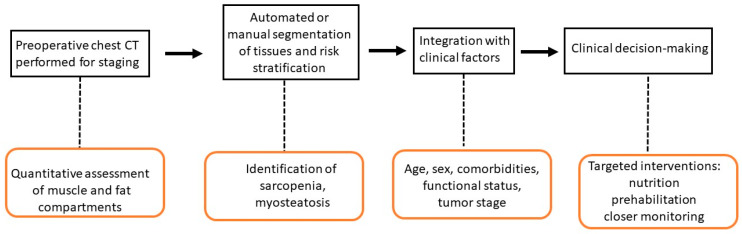
CT-Based Body Composition for Postoperative Risk Stratification in Lung Cancer Surgery.

Enhanced Recovery After Surgery (ERAS)-based protocols that integrate both exercise and nutrition have demonstrated improvements in nutritional markers (albumin, prealbumin), muscle mass, and functional capacity, with a trend toward lower complication rates and faster recovery [48]. ERAS protocols play a central role in improving perioperative outcomes for lung cancer patients undergoing thoracic surgery. Key elements include early mobilization, multimodal opioid-sparing analgesia, early oral nutrition, judicious fluid management, and minimization of invasive monitoring [Table 2] [Figure 1].

Implementation of ERAS protocols in lung cancer surgery is associated with significantly reduced postoperative complications (including pulmonary, cardiac, and surgical site infections), shorter hospital length of stay, decreased opioid use, and lower direct costs, without increasing readmission or mortality rates [49].

Early removal of chest tubes and urinary catheters, as well as increased rates of minimally invasive surgery, further contribute to improved outcomes.

## 7. The Role of CT-Based Body Composition in Other Diseases

CT-based body composition analysis has been utilized for risk evaluation in various conditions beyond lung cancer, with both case reports and extensive cohort studies validating its prognostic significance. In colorectal cancer, automated CT measurements of skeletal muscle attenuation, subcutaneous and visceral fat, and aortic calcium have been linked to overall survival prediction. Individuals with lower muscle attenuation and reduced subcutaneous fat area on pre-treatment abdominal CT exhibited significantly increased mortality risk, regardless of BMI. Combining muscle attenuation, subcutaneous fat, and aortic calcium improved risk classification compared with conventional indicators [50]. Studies comparing colorectal and lung cancer populations further confirm that CT-derived muscle and fat metrics are prognostic across different tumor types, reflecting overall patient health and systemic inflammation rather than disease stage alone [51]. In cardio-metabolic disorders, abdominal CT scan opportunistically provide automated assessments of visceral fat, muscle volume, and aortic calcium, which are associated with future cardiovascular events and mortality. When used together, these CT biomarkers perform on par with—or better than—established clinical models for predicting outcomes such as myocardial infarction, heart failure, and fragility fractures [52]. For chronic and aging-related diseases, population-level research has defined normative ranges for CT-based measures of muscle area, muscle density, and fat distribution. These biomarkers correlate with the presence and severity of chronic conditions like diabetes, cardiovascular disease, and cirrhosis, and they predict mortality and functional decline even in asymptomatic adults [53].

Case series and large-scale cohort studies indicate that individuals with multiple coexisting conditions, reduced functional status, or those scheduled for high-risk operations (such as vascular, thoracic, or advanced gastrointestinal oncologic surgery) face a markedly higher likelihood of major postoperative complications and death. A retrospective analysis including more than 10 million non-cardiac surgical procedures reported a 3.0% incidence of the composite outcome of perioperative mortality, myocardial infarction, and ischemic stroke, while myocardial injury was observed in as many as 20% of cases [54]. In the setting of complex gastrointestinal cancer surgery, serious adverse events—including anastomotic failure and sepsis—remain common, and systematic reviews have identified a wide range of modifiable preoperative risk factors suitable for targeted perioperative optimization strategies [55]. Preoperative risk evaluation is therefore a critical component of surgical care, enabling the identification of high-risk patients, supporting individualized perioperative planning, and facilitating shared decision-making. Robust risk stratification allows for personalized interventions such as pre-habilitation programs, coordinated multidisciplinary care, and intensified postoperative monitoring, all of which may reduce complication rates and improve clinical outcomes [56].

## 8. Artificial Intelligence in CT-Derived Body Composition Analysis

Artificial intelligence (AI) has significantly transformed CT-based body composition assessment by enabling fully automated, fast, and highly precise segmentation and quantification of tissues, including skeletal muscle, visceral and subcutaneous fat, and bone structures. Deep learning approaches—most notably convolutional neural networks—have shown outstanding performance, with Dice similarity coefficients often exceeding 0.9 for segmentation tasks, achieving accuracy comparable to or even surpassing that of expert manual annotations in both single-slice and three-dimensional analyses [57].

These automated methods support opportunistic screening and risk stratification across oncologic and non-oncologic populations by utilizing routinely acquired CT imaging performed for other clinical purposes. Moreover, AI-driven solutions substantially decrease the time and effort required for image analysis, thereby enabling large-scale studies and facilitating incorporation into routine clinical practice. Despite these advances, important challenges persist, including the need for standardization, robust external validation, and the definition of normative reference values and clinically relevant thresholds. Current research efforts are therefore directed toward multicenter validation and the development of consensus standards for accuracy and precision to enable broader clinical implementation [58].

## 9. Conclusions

CT-based opportunistic assessment of body composition has emerged as a powerful, objective tool for preoperative risk stratification in lung cancer surgery, offering significant advantages over traditional clinical measures, such as BMI.

The integration of automated artificial intelligence-based segmentation algorithms enables rapid, reproducible quantification of muscle mass, muscle quality, and adipose tissue compartments from routine staging CT scans, facilitating seamless incorporation into clinical workflows without additional imaging or patient burden.

This opportunistic approach transforms existing diagnostic imaging into a multidimensional assessment of physiologic reserve, identifying sarcopenia, myosteatosis, and sarcopenic obesity—conditions that are highly prevalent yet frequently undetected by conventional screening methods.

CT-based body composition measurements—especially skeletal muscle index and muscle radiodensity—are independently associated with postoperative complications and prolonged hospital stays following lung cancer surgery. These parameters provide more accurate risk prediction than traditional indicators such as BMI or frailty scales, offering objective, reproducible data that can be obtained opportunistically from routine preoperative imaging. Assessing muscle quality (via radiodensity) and identifying sarcopenic obesity further enhance prognostic accuracy, and sex-related variations in muscle and fat distribution may also affect complication risk. Automated and semi-automated CT analysis allows these assessments to be quickly incorporated into clinical practice, supporting personalized risk stratification and potentially informing prehabilitation and perioperative care. This approach is practical using standard thoracic CT scans obtained for lung cancer staging and shows good agreement with established lumbar-based measurements.

Future directions include standardization of measurement protocols across thoracic vertebral levels, validation of sex-specific and population-specific cutoff values, prospective evaluation of intervention strategies guided by CT metrics, and full integration of automated body composition reporting into radiology workflows and electronic health records to ensure widespread clinical implementation. Future efforts will focus on harmonizing technical acquisition and analysis parameters, establishing consensus standards for accuracy and precision, and developing large multicenter datasets to support robust algorithm training and validation. Emerging CT technologies, including dual-energy and photon-counting CT, hold promise for improved tissue characterization; however, standardization across platforms will be essential to maintain reproducibility. Ultimately, the shift from research applications to routine clinical use is anticipated to facilitate the opportunistic derivation of prognostic biomarkers from standard CT imaging, enabling personalized treatment strategies and refined risk stratification, particularly in oncology, cardiometabolic disorders, and perioperative medicine [59].

## Figures and Tables

**Table 1:** Key Variables Assessed by CT-Based Body Composition Analysis in Lung Cancer Surgery.

Variable	Definition	Clinical Relevance
Skeletal Muscle Index (SMI)	Cross-sectional muscle area at T10/T12/L3, normalized by height	Quantifies muscle mass
Skeletal Muscle Density (SMD)	Mean muscle attenuation in Hounsfield units at T10/T12/L3	Assesses muscle quality
Visceral Adipose Tissue (VAT)	Cross-sectional area or volume of intra-abdominal fat at T10/T12/L3	VAT volume inversely associated with recurrence risk
Subcutaneous Adipose Tissue (SAT)	Cross-sectional area or volume of subcutaneous fat at T10/T12/L3	Higher SAT density linked to increased recurrence risk
Intermuscular Adipose Tissue (IMAT)	Fat area within and between muscle groups at T10/T12/L3	Marker of muscle quality (myosteatosis)
Pectoral Muscle Index (PMI)	Area of pectoral muscle at chest level, normalized by height	Alternative muscle mass measure on chest CT
Paravertebral Muscle Index (PVMI)	Area of paravertebral muscle at chest level, normalized by height	Alternative muscle mass measure; low PVMI associated with poor survival

**Table 2:** Summary of Key Nutritional Variables in Perioperative Care for Surgery within Enhanced Recovery After Surgery (ERAS) Protocols.

Nutritional Variable	Role in Surgery ERAS Protocols
Nutritional risk screening	Identify patients at risk for malnutrition
Preoperative fasting duration	Minimize catabolic stress
Carbohydrate loading	Reduce insulin resistance
Protein intake	Preserve muscle mass
Energy intake	Support healing
Early oral feeding	Accelerate gut recovery
Oral nutritional supplements	Address increased needs
Immunonutrition	Modulate inflammation
Glycemic control	Prevent hyperglycemia-related complications
Micronutrient status	Support immune function
Fluid management	Prevent ileus

## Data Availability

No new data were generated during this review.

## Abbreviations

The following abbreviations are used in this manuscript:

CT	Computed tomography
NSCLC	Non-small-cell lung cancer
BMI	Body mass index
SMI	Skeletal muscle index
GLIS	Global leadership initiative in sarcopenia
VAT	Visceral adipose tissue
SAT	Subcutaneous adipose tissue
BIA	Bioelectrical impedance analysis
DXA	Dual-energy X-ray absorptiometry
GLIM	Global Leadership Initiative on Malnutrition
SMD	Skeletal muscle density
COPD	Chronic obstructive pulmonary disease
DLCO	Diffusing capacity for carbon monoxide
ERAS	Enhanced Recovery After Surgery
IMAT	Intermuscular adipose tissue
PMI	Pectoral muscle index
PVMI	Paravertebral muscle index
RCRI	Revised cardiac risk index
ASA	American Society of Anaesthesiology
NSQIP	National Surgical Quality Improvement Program
